# Evaluation of logistic Bayesian LASSO for identifying association with rare haplotypes

**DOI:** 10.1186/1753-6561-8-S1-S54

**Published:** 2014-06-17

**Authors:** Swati Biswas, Charalampos Papachristou

**Affiliations:** 1 Department of Mathematical Sciences, FO 35, University of Texas at Dallas, 800 West Campbell Road,Richardson, TX 75080, USA; 2 Department of Mathematics, Physics, and Statistics, University of the Sciences in Philadelphia, 600 South 43rd Street, Philadelphia, PA 19104, USA

## Abstract

It has been hypothesized that rare variants may hold the key to unraveling the genetic transmission mechanism of many common complex traits. Currently, there is a dearth of statistical methods that are powerful enough to detect association with rare haplotypes. One of the recently proposed methods is logistic Bayesian LASSO for case-control data. By penalizing the regression coefficients through appropriate priors, logistic Bayesian LASSO weeds out the unassociated haplotypes, making it possible for the associated rare haplotypes to be detected with higher powers. We used the Genetic Analysis Workshop 18 simulated data to evaluate the behavior of logistic Bayesian LASSO in terms of its power and type I error under a complex disease model. We obtained knowledge of the simulation model, including the locations of the functional variants, and we chose to focus on two genomic regions in the *MAP4 *gene on chromosome 3. The sample size was 142 individuals and there were 200 replicates.

Despite the small sample size, logistic Bayesian LASSO showed high power to detect two haplotypes containing functional variants in these regions while maintaining low type I errors. At the same time, a commonly used approach for haplotype association implemented in the software hapassoc failed to converge because of the presence of rare haplotypes. Thus, we conclude that logistic Bayesian LASSO can play an important role in the search for rare haplotypes.

## Background

It is now widely acknowledged that rare variants play a critical role in complex diseases. Although many approaches have been proposed for detecting association with rare single-nucleotide variants (eg, Refs. [1-6] to name just a few), there are relatively fewer approaches for rare haplotype variants [7-10]. Once a particular genomic region is implicated to be potentially harboring a functional variant from single nucleotide polymorphism (SNP) analysis, typically it is followed up by haplotype analysis to zoom further into the region. In such analysis, rare haplotypes frequently surface because rare haplotypes can result from even combinations of common single variants.

The presence of rare haplotypes poses a challenge for commonly used haplotype association approaches such as those based on generalized linear models using the expectation-maximization (EM) algorithm (implemented in the software hapassoc [11] among others). With rare haplotypes, EM estimates can be unstable and the algorithm may fail to converge. To circumvent this problem, rare haplotypes are usually pooled together. However, pooling can result in washing out of association signal if haplotypes of risk and protective types are pooled together [10]. Thus, in recent years, newer approaches for detecting rare haplotypes have been proposed. One of them is logistic Bayesian LASSO (LBL) [10], a Bayesian version of penalized regression. LBL applies penalty to regression coefficients through appropriate choice of their prior distributions. This helps reduce signal noise by weeding out unassociated (especially common) haplotypes, thereby enabling signals contained in the associated rare haplotypes to be more easily detected. For example, the application of LBL to age-related macular degeneration data led to identification of a specific rare haplotype for the first time in the literature [10].

Our goal is to further evaluate the performance of LBL for data generated under complicated and realistic scenarios. Data from Genetic Analysis Workshop 18 (GAW18) provide such an opportunity, and with this aim, we apply LBL to 200 replicates of the simulated GAW18 data. In particular, we mimic a candidate variant search approach. That is, we assume that prior studies, most likely single-SNP studies, have pointed to a genomic region that potentially harbors variants involved in the genetic mechanism of a trait. Following up on that, we zoom into the region with sequence data provided in GAW18. So that we could evaluate power and type I error, we obtained access to the simulating model ("Answers"). We focused on two genomic regions in the *MAP4 *gene on chromosome 3 that harbored several functional variants and analyzed them using LBL and hapassoc.

## Methods

Here we briefly describe LBL; more details can be found in Biswas and Lin [10]. Suppose we have a case-control sample consisting of *n*_1_ cases and *n*_2_ controls with *n*_1 _+ *n*_2 _= *n*. Let *Y_i _= *1/0 denote the case-control status of the *i*^th^ individual, *i *= 1*. . .n*, and ***Y ***= (*Y*_1_*,...,Y_n_*). Suppose *L *SNPs are considered to form a haplotype block. We further let *Z_i _*denote the missing (phased) haplotype pair of *i*^th ^individual and ***Z = ***(*Z*_1_*. . . Z_n_*). Note that *Z_i_*s are unobservable because phase information is usually not deductible from the genotype data. LBL is based on retrospective model for case-control data, which has been well studied in general statistics (including Bayesian), as well as in haplotype-association literature (see Refs. [12-16] and the references therein). The complete data likelihood is written as:

(1) $$L_{c}(\Psi )=\prod_{i=1}^{n_{1}}P(Z_{i}|Y_{i}=1,\Psi )\prod_{i=n_{1}+1}^{n}P(Z_{i}|Y_{i}=0,\Psi )$$

where Ψ=(***β***,***γ***) denotes the collection of regression coefficients and parameters associated with haplotype frequencies, which will be specified more explicitly as our formulation unfolds. Let *a_Z_*=*P*(*Z|Y *= 0) and *b_Z_*=*P*(*Z|Y *= 1) denote the frequencies of a haplotype pair *Z *in the control and the case population, respectively. We first note that we can express *b_Z _*in terms of *a_Z _*and the odds of disease for a given *Z, θ_Z_*:

$$b_{Z}=\frac{P\left(Y=1\text{|}Z\right)P\left(Z\right)}{\sum_{H}P\left(Y=1\text{|}H\right)P\left(H\right)}=\frac{\theta_{Z}P\left(Z\right)P\left(Y=0\text{|}Z\right)}{\sum_{H}\theta_{H}P\left(H\right)P\left(Y=0\text{|}H\right)}=\frac{\theta_{Z}a_{Z}}{\sum_{H}\theta_{H}a_{H}}$$

where *θ_Z_*=*P*(*Y*=1*|Z*)*/P*(*Y*=0*|Z*), and *H *is the set of all possible haplotype pairs. Therefore, the likelihood in equation (1) can be expressed in terms of the *a_Z _*s and the *θ_Z_*s. Let us next consider *a_Z_* and *θ_Z_*, and specify their models.

### Modeling of *a_Z_*

Suppose there are a total of *m* haplotypes (ie, haplotype diversity is *m*) and let ***f ***= (*f*_1_*,...,f_m _*) denote their frequencies with the constraint that *f_k_>*0 and $\sum_{k=1}^{m}f_{k=1}$. Then, for a haplotype pair *Z*= *z_k_/z_k ′ _*, we can model *a_Z _*as follows:

(2) $$a_{Z}\left(\gamma \right)=P\left(Z=z_{k}/{z_{k}}_{\prime }|Y=0,\gamma \right)=\delta_{kk\prime }df_{k}+\left(\text{2}-{\delta_{kk}}_{\prime }\right)\left(1-d\right)f_{k}{f_{k}}_{\prime }$$

where *δ_kk ′ _*= 1(0) if *z_k _*=*z_k′ _* (*z_k _≠ z_k′_*), ***γ ***={***f****,d*} and *d*∈(*−*1,1) is the within-population inbreeding coefficient that can be used to capture excess and/or reduction of homozygosity. By modeling the frequency in this way, we do not need to make the assumption of Hardy-Weinberg equilibrium.

### Modeling of *θ_Z_*

We use logistic regression for modeling log odds. Specifically, log *θ_Z _=α *+ ***X***_*Z*_***β***, where ***X***_*Z *_is a (row) design vector, *α *is the intercept, and ***β ***is a vector of coefficients representing the haplotype effects.

### Priors

To cast the problem in the Bayesian setting, we need to assign priors to the parameters Ψ = (***β***, ***γ ***= {***f****, d*}). The prior for *β *plays the important role of regularization of regression coefficients. In particular, a double-exponential distribution with mean 0 and appropriately chosen variance to control the degree of penalty has been shown to give the Bayesian version of LASSO when normal likelihood is used. Specifically, we set the prior for *β_j _*to be

(3) $$\pi (\beta_{j}|\lambda )=\frac{\lambda }{2}\text{exp}\left(-\lambda \left|\beta_{j}\right|\right),-\infty <\beta_{j}<\infty ,j=1,\ldots ,m-1.$$

Here *λ *controls the degree of penalty as the variance of this distribution is 2*/λ*^2^. We let the hyper-parameter *λ *follow a gamma distribution. For ***f ***and *d*, note that they are not independent, as *a_Z_*(***γ***) in equation (2) must be nonnegative. This imposes the constraint that *d >*{*−f_k_/*(1 *− f_k_*)} for all *k*, and because *d <*1, we have max *_k_*{*−f_k_/*(1 *− f_k_*)}*<d <*1. We use uniform priors for both ***f ***and *d *in their restricted ranges. For ***f***, we use Dirichlet(1, 1*, ...*, 1) consisting of a total of *m *1s for the *m *haplotypes; for *d *given ***f***, we use the uniform(max *_k_*{*−f_k_/*(1 *− f_k_*)}, 1) distribution.

### Inference

Markov chain Monte Carlo (MCMC) methods are used to estimate posterior distributions of parameters. At each iteration, we update the missing haplotypes ***Z ***and the parameters ***β***, *λ*, ***f***, and *d*. The Markov chain is run for a total of 50,000 iterations with 20,000 burn-in. We draw an inference regarding association by testing for significance of each *β *coefficient. We carry out a hypothesis test of *H*_0 _: *|β| ≤ ∈ *versus *H_a_*: *|β| >∈*, where *∈* is set to a small number, using Bayes factor (BF), which is defined as the ratio of posterior odds to prior odds of *H_a_*. If the BF exceeds a certain threshold, we conclude that the corresponding *β *is significant;that is, that haplotype is associated. In our applications, we use a threshold of 2 and *∈*= 0.1 following Biswas and Lin [10]. This method has been implemented as an R package (with dynamic loading of C program) LBL, which is available at http://www.utdallas.edu/~swati.biswas.

LBL requires as an input a list of possible haplotypes that are compatible with each person's genotype. We obtain this from the hapassoc package's pre-processing command "pre.hapassoc". Note that LBL does not assign any specific haplotype to persons whose haplotypes cannot be inferred unambiguously; rather, their haplotypes are treated as missing and updated at every MCMC iteration. Thus, the uncertainty in haplotypes for each person is incorporated into the model. By the same token, the uncertainty in haplotype frequencies *f_k_* is taken into account by treating them as unknown parameters with noninformative prior distribution and updating them at each iteration (note that although pre.hapassoc outputs frequency estimates also, those are not needed by LBL). So, even if in the data some haplotypes are only compatible with the cases but not with any control, by having a prior distribution on *f_k_*s, the model allows the possibility of the control population having those haplotypes as well. However, if those haplotypes have sufficient contribution to the disease under study and the sample size is reasonable, we will expect those haplotypes to be inferred as associated.

## Results

As mentioned in the background section, we propose to use haplotype analysis as a follow-up analysis to zoom into a genomic region that has been implicated in earlier studies. With this premise, we focus on gene *MAP4 *on chromosome 3. In particular, we consider 2 separate regions around 2 functional variants: (a) the most frequent functional variant located at 47,956,424 basepairs (bp) with minor allele frequency (MAF) =0.378, and (b) variant with strongest effect size at 48,040,283 bp with MAF = 0.032. The rationale for such a choice is that a functional variant that is either fairly common or is rare but has a strong effect could be reasonably expected to have been implicated in prior (single-SNP genome scan) studies. To zoom into these regions, we selected SNPs that are within 4000 bp of these locations and have MAF>0.01, and formed haplotypes with those SNPs in each region. This choice of MAF ensured that in a sample of size 142, at least two copies of each SNP were present and thereby excluded almost monomorphic SNPs. Common SNPs can combine to form rare haplotypes (frequency <0.05), and, indeed, this was the case for these regions. In both regions, we had 9 SNPs each. Specifically, for region 1, 9 SNPs resulted in 9 haplotypes. This is unusually low and it could be just random chance or there may be a biological reason for this. However, as we analyze the simulated phenotypes, which were generated based on single SNPs, we do not expect this to have any significant effect on our analysis.

As LBL has been proposed for the case-control data, we used the sample of unrelated people. We classified a person as affected (case) if the individual was diagnosed with high blood pressure or was taking medication for high blood pressure at the last exam. The total sample size was 142 individuals. The number of cases varied from replicate to replicate with mean = 72 and SD = 5, while the genotypes were identical for all replicates. We analyzed all 200 replicates for the two regions described above twice. First, we analyzed with the provided phenotypes to examine power. Second, for each replicate, we randomly permuted the affection status of individuals and reanalyzed the data. This nullified any association present between phenotype and genotypes, and thereby allowed us to gauge the type I error. Note that the total numbers of cases and controls remain the same in both the original and its corresponding "null" versions.

We analyzed each replicate using hapassoc and LBL. Hapassoc did not converge, presumably because of the presence of rare haplotypes in both regions studied. Tables 1 and 2 show SNPs and haplotypes in these two genomic regions and the proportions of replicates showing association with each haplotype (BF>2) in both original and null versions. In Table 1 LBL's power to detect the haplotype with two functional variants (the third functional variant has almost a negligible effect) is exceptionally high for a sample size of only 142 individuals. However, there is no power at all to identify the haplotypes with 1 functional variant at 47,956,424 bp. This indicates that the other (rare) functional variant at 47,957,996 bp almost solely drives the power for detecting the haplotype with two variants. The type I errors in the null version are all very low (≤2%). Thus, this illustrates the power of LBL for identifying rare haplotypes. In region 2, the powers are 78% and 30% for the two haplotypes; both are rare haplotypes and each contains one variant. The variant in the former haplotype accounts for higher percentage of the variability in the diastolic blood pressure (0.02 vs. 0.01) and systolic blood pressure (0.03 vs. 0.01). Although the two variants are almost on top of each other (1 bp apart), there was no haplotype containing both variants. The type I errors are, again, very small (≤2%).

**Table 1:** Results for the region surrounding SNP at 47,956,424 bp using phenotypes as provided (Original) and after randomly redistributing them to individuals (Null).

SNP(bp)	MAF	Hap1	Hap2	Hap3	Hap4	Hap5	Hap6	Hap7	Hap8	Hap9
47,952,843	0.018	0	0	0	0	0	0	0	1	1
47,953,405	0.370	0	1	1	1	1	1	1	1	1
47,953,733	0.320	0	0	0	0	1	1	1	0	0
47,956,424	0.359	0	0	**1**	**1**	**1**	**1**	**1**	**1**	**1**
47,956,506	0.317	0	0	0	0	0	1	1	0	0
47,957,996	0.021	0	0	0	0	0	0	**1**	0	0
47,958,037	0.317	0	0	0	0	0	**1**∗	**1**∗	0	0
47,959,770	0.367	0	1	1	1	1	1	1	0	1
47,959,977	0.011	0	0	0	1	0	0	0	0	0
Hap Freq		0.63	0.011	0.011	0.011	0.003	0.296	0.021	0.007	0.011
Original		NA	0.00	0.00	0.00	0.00	0.00	0.89	0.00	0.00
Null		NA	0.00	0.00	0.00	0.00	0.00	0.02	0.00	0.00

The two rows, Original and Null, show the proportion of replicates with BF >2. There are 9 possible haplotypes (Hap) with minor allele of each SNP denoted by 1. The minor alleles in bold typeface are functional variants. The most frequent haplotype (Hap1) is the baseline. Freq represents haplotype frequency.

^∗^The % variability explained by this variant is almost negligible (<0.0001).

**Table 2:** Results for the region surrounding SNP at 48,040,283 bp using phenotypes as provided (Original) and after randomly redistributing them to individuals (Null).

SNP(bp)	MAF	Hap1	Hap2	Hap3	Hap4	Hap5	Hap6	Hap7	Hap8	Hap9	Hap10	Hap11	Hap12
48,036,889	0.011	0	0	0	0	0	0	0	0	0	0	1	1
48,037,078	0.363	0	0	0	0	1	1	1	1	1	1	0	0
48,038,714	0.373	0	0	0	0	1	1	1	1	1	1	1	1
48,039,908	0.246	0	1	1	1	0	0	0	0	0	0	0	0
48,040,283	0.025	0	0	0	0	0	0	0	0	0	**1**	0	0
48,040,284	0.021	0	0	0	**1**	0	0	0	0	0	0	0	0
48,041,471	0.025	0	0	0	0	0	0	1	1	1	0	0	1
48,042,192	0.018	0	0	0	0	0	0	0	1	1	0	0	1
48,043,058	0.331	0	0	1	0	0	1	0	0	1	1	0	0

Hap Freq		0.380	0.222	0.004	0.021	0.018	0.299	0.007	0.010	0.004	0.025	0.007	0.003

Original		NA	0.02	0.00	0.30	0.02	0.00	0.00	0.00	0.00	0.78	0.00	0.00
Null		NA	0.01	0.00	0.00	0.02	0.01	0.00	0.00	0.00	0.00	0.00	0.00

The two rows, Original and Null, show the proportion of replicates with BF >2. There are 12 possible haplotypes (Hap) with minor allele of each SNP denoted by 1. The minor alleles in red and boldface are functional variants. The most frequent haplotype (Hap1) is the baseline. Freq represents haplotype frequency.

## Discussion

Here we evaluated the power and type I error of LBL for detecting rare haplotypes. Remarkably, even with a sample size of 142 individuals, LBL seems to be able to achieve very high power and at the same time hold the type I error at very low levels. Even though this high power may be partially explained by the relatively large contribution of the variants to the trait variance (in the range of 0.01% to 0.03%), these results further strengthen the earlier findings on the usefulness of LBL [10].

LBL is relatively computationally efficient. To run one replicate for region 1 (with nine haplotypes), LBL took approximately eight seconds on a 2.8GHz Xeon processor under a Linux operating system with 23.5 GB of RAM. The computational intensity depends on the number of haplotypes rather than the number of SNPs. In our experience, LBL can easily handle 15 to 20 haplotypes. Also, haplotype frequencies of 0.005 were handled well by LBL in Ref. [10]. We also expect LBL to handle rarer haplotypes with larger sample sizes.

The nature of the simulating model and the resulting data, although complex, lead to some limitations of the study. First, the simulation model was based on individual SNPs and not haplotypes, whereas from a biological point of view, the role of haplotypes is more than just a combination of SNPs [17]. Our limited exploration of the data (including other regions not presented here) indicated that there may be only few haplotypes that consisted of more than one functional variant. Our first region may be one of the few examples of that type. Even for that haplotype, as we saw earlier, the power to detect association with it seemed to be solely driven by one of the variants. Furthermore, in our second region, from a biological standpoint, it would seem highly unlikely that two *independent *mutations would have arisen so close to each other (there was no haplotype with both mutations) [17].

A second limitation is the sample size of only 142. With such a small sample, detecting association with a rare variant is highly unlikely. Thus, we chose variants with relatively stronger effects. Nevertheless, it is noteworthy that LBL showed reasonable powers when hapassoc failed to converge. It will be of interest to compare the results with some newer methods for rare haplotype association.

Yet another shortcoming of the current study is that the simulated genotypes in all replicates were the same and only phenotypes varied across replicates. For this reason, we did not combine replicates to increase the sample size and explore power for detecting haplotypes with more modest effects. Nonetheless, it has been shown that LBL has good power for detecting such type of effects with larger sample sizes [10]. All together, the results establish LBL as a powerful tool for identifying association with rare haplotypes. Moreover, the flexible framework of LBL allows many useful extensions. For example, covariates and their interactions with haplotypes can be incorporated by modeling log *θ_Z _*= *α *+ ***X***_*Z*_***β ***+ ***X***_*E*_**τ + X**_Z _**X**_E_***γ ***with ***X***_**E **_denoting the design vector based on covariates, ***τ ***the corresponding coefficients, and ***γ ***the coefficients corresponding to interactions. This extension has been recently proposed in Ref [18]. For this, the likelihood in equation (1) is extended to model the joint distribution of haplotypes and covariates. Another future work is to adapt LBL for other data types.

## Conclusions

LBL is a powerful approach for detecting rare haplotype association.

## Competing interests

The authors declare that they have no competing interests.

## Authors' contributions

SB and CP designed the overall study and conducted statistical analyses. SB drafted the manuscript and CP helped in its revision. All authors read and approved the final manuscript.
